# A case report and literature review on reactive cutaneous capillary endothelial proliferation induced by camrelizumab in a nasopharyngeal carcinoma patient

**DOI:** 10.3389/fonc.2023.1280208

**Published:** 2023-11-28

**Authors:** Yao Lin, Yuxin Lin, Xiaoping Zhong, Qingshan Chen, Shijie Tang, Jiasheng Chen

**Affiliations:** Department of Plastic Surgery and Burn Center, Second Affiliated Hospital, Shantou University Medical College, Shantou, Guangdong, China

**Keywords:** reactive cutaneous capillary endothelial proliferation, camrelizumab, nasopharyngeal carcinoma, case report, literature review

## Abstract

Camrelizumab, a monoclonal antibody, blocks programmed cell death protein-1 from binding to T cells and programmed cell death ligand 1 on tumor cells, thereby ensuring sustained T cell activation and blocking immune escape of various types of cancer, including nasopharyngeal carcinoma. Reactive cutaneous capillary endothelial hyperplasia (RCCEP) is the most common immune-related adverse event in patients treated with camrelizumab. We report a case nasopharyngeal carcinoma in a patient with camrelizumab-induced RCCEP. A 68-year-old man diagnosed with nasopharyngeal carcinoma developed RCCEP at multiple locations after 3 months of camrelizumab treatment. RCCEP of the right lower eyelid affected closure of the right eye. In this report, we also reviewed previous literature on camrelizumab-induced RCCEP. In summary, the mechanism underlying camrelizumab-induced RCCEP remains unclear. RCCEP typically gradually subsides after discontinuing camrelizumab treatment. Larger nodules can be treated with lasers, ligation, or surgery. Although surgical excision is effective, RCCEP may recur in patients undergoing camrelizumab treatment. RCCEP management may not be required in the absence of adverse effects on the patient’s daily life.

## Introduction

In recent years, immune checkpoint blockade therapy has demonstrated remarkable efficacy in the treatment of various malignant tumors (1, 2). Previous studies have shown that programmed cell death ligand 1 is highly expressed in patients with nasopharyngeal carcinoma (NPC) (3–5). Therefore, the combination of programmed cell death protein 1 and programmed cell death ligand 1, which are expressed by T cells and tumor cells, respectively, can block signal transduction and enhance immune system activity, thereby destroying cancer cells (6, 7). Camrelizumab is a monoclonal antibody against programmed cell death protein 1 that was developed by Jiangsu Hengrui Medicine Co (8).

Immune checkpoint inhibitors are associated with a range of immune-related adverse events (irAEs) (9, 10), which are often associated with immune system overactivation. Reactive cutaneous capillary endothelial hyperplasia (RCCEP) is the most common adverse event associated with camrelizumab use and usually occurs in the skin of the head, face, and trunk (11). However, the mechanism underlying camrelizumab-induced RCCEP remains unclear. We herein present a case in which a patient with NPC developed RCCEP, a “tumor-like” nodule, on the right lower eyelid after undergoing camrelizumab and chemotherapy. RCCEP uncommonly occurs in this location, and the nodule interfered with the patient’s ability to close the right eye owing to the thinness of the skin in this area. The nodule was surgically removed, and the patient’s prognosis was good.

## Case report

**Figure 1:**
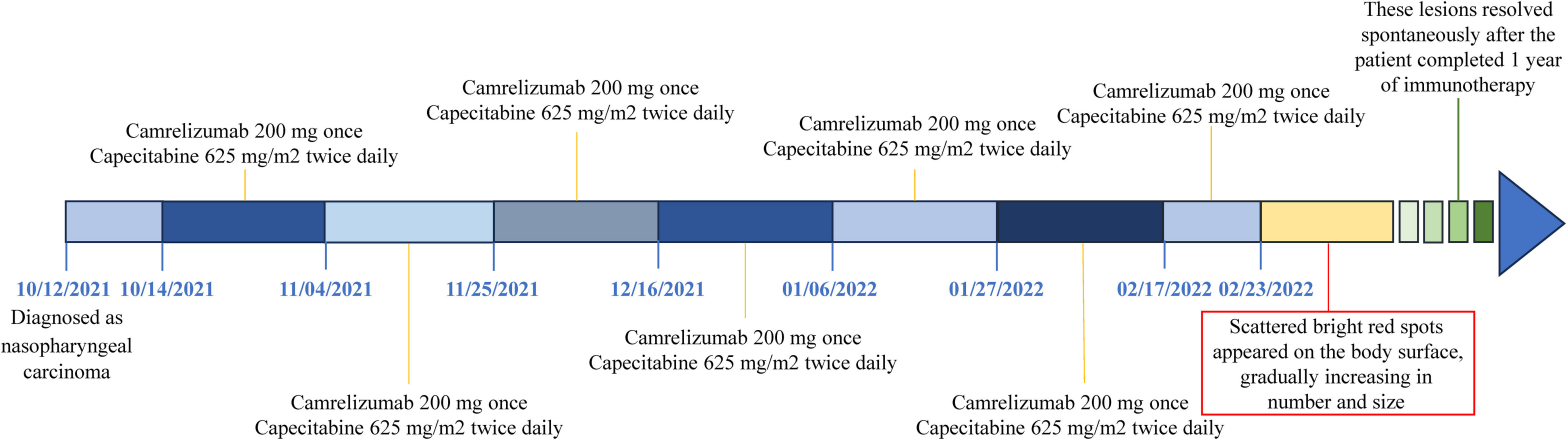
The timeline of patient progress throughout treatment.

**Figure 2:**
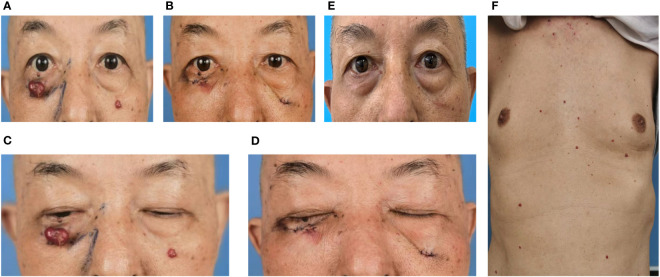
Dark red nodules were distributed over the patient’s head, face, and trunk. **(A, C)** Before surgery. **(B, D)** After surgery. **(C)** Before surgery, the nodule pulled on the right lower eyelid, resulting in incomplete closure of the right eye. **(D)** After surgery, the patient could close the right eye normally. **(E)** Six months after the operation, no recurrence of the right lower eyelid nodule was observed, although new nodules were noted in other parts of the face. **(F)** Dark red nodules distributed over the patient’s trunk before surgery.

**Figure 3:**
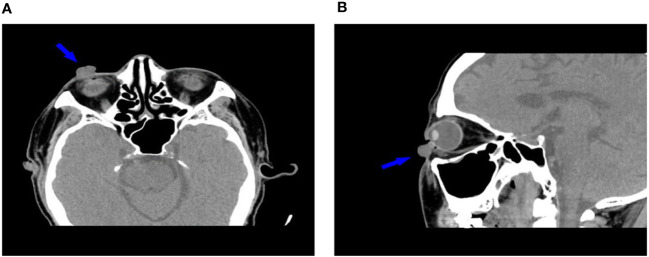
Head computed tomography (**A**: transverse plane, **B**: sagittal plane) shows the relationship between the nodule and surrounding soft tissues (arrows).

In October 12, 2021, a 68-year-old man was diagnosed with T3N2M1 NPC, based on the American Joint Committee on Cancer’s Cancer Staging Manual, Eight Edition (12). The patient received chemotherapy (capecitabine, 625 mg/m2 twice daily, orally) and immunotherapy (camrelizumab injection, 200 mg every 21 days). On February 17, 2022, the patient underwent the seventh cycle of camrelizumab injection therapy. The timeline of the patient’s entire treatment progress is shown in **Figure 1**. Approximately 6 days later, the patient developed scattered bright red spots on the head, face, and trunk. Some spots gradually developed into pea-sized nodules (**Figure 2**). Two months later, the nodule on the right lower eyelid had grown to the size of a peanut (**Figure 2**). Head computed tomography revealed a nodule that protruded outward and squeezed the normal eye tissue inward (**Figure 3**). In addition, the nodule pulled the lower eyelid downwards, which affected eyelid closure owing to the thin skin and soft tissue of the lower eyelid (**Figures 2A, C**). After considering the patient’s condition and preferences, we resected the right lower eyelid nodule. The patient recovered well postoperatively and was able to close the right eye without difficulty (**Figures 2B, D**).

**Figure 4:**
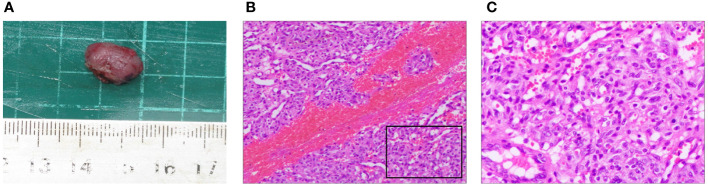
Pathological characteristics of the right lower eyelid nodule. **(A)** The size of the surgically resected nodule was about 1.5 cm × 1.0 cm × 0.9 cm. **(B, (C)** Hematoxylin and eosin staining showed extensive capillary proliferation. **(B)** × 200; **(C)** × 400.

The removed nodule is shown in (**Figure 4A**). Histopathological examination revealed that the lesions comprised proliferated capillaries, which were distributed in nodular and lobulated forms. Large vessels were surrounded by small vessels; lumens varied in size and contained red blood cells (**Figure 4B**). Vascular endothelial cells were densely arranged. The nuclei were oval or short and spindle-shaped; mitotic figures were easily observed (**Figure 4C**). These pathological results supported a diagnosis of RCCEP. During the 6-month follow-up period, the surgical area of the right lower eyelid had recovered well, without RCCEP recurrence (**Figure 2E**). Because the patient continued to receive camrelizumab postoperatively, dark red nodules remained on other parts of the body (**Figure 2E**); however, these lesions resolved spontaneously after the patient completed 1 year of immunotherapy. According to Naranjo’s adverse drug reaction probability scale (**Table 1**), RCCEP was most likely caused by camrelzumab.

**Table 1:** Naranjo’s adverse drug reaction probability scale.

Related issues	results	score
1. Are there previous conclusive reports of this reaction?	yes	+1
2. Did adverse event appear after the suspected drug was given?	yes	+2
3. Did the adverse reaction improve when the drug was discontinued or a specific antagonist was given?	yes	+1
4. Is the ADR repeated after the use of the suspected drug again?	not known	0
5. Are there alternative causes that could have caused the reaction?	no	+2
6. Did the reaction reappear when a placebo was given?	not known	0
7. Was the drug detected in any body fluid in toxic concentrations?	not known	0
8. Was the reaction more severe when the dose was increased, or less severe when the dose was decreased?	not known	0
9. Did the patient have a similar reaction to the same or similar drugs in any previous exposure?	no	0
10. Was the adverse event confirmed by any objective evidence?	no	0
Total score		6

Naranjo’s score ≥ 9 points: definite, 5-8 points: probable, 1-4 points: possible, ≤ 0 points: doubtful.

## Discussion

### RCCEP occurrence after camrelizumab administration

Skin reactions in various organs are the most frequent irAEs, which are triggered by immune checkpoint inhibitors (13). The most frequent side effect of camrelizumab is RCCEP (8, 11). RCCEP appears primarily in the skin of the head, face, and torso (11). RCCEP in these regions is not typically fatal, although may affect function and coordination in the affected regions. The present case involved a patient who received camrelizumab therapy and subsequently developed right lower eyelid RCCEP that affected the patient’s ability to close the eye. According to current RCCEP grading criteria, this patient was classified as having a grade 2 lesion (single or multiple nodules, with the greatest nodule diameter being >10 mm, with or without rupture and bleeding) (14). Surgery was performed to restore the patient’s ability to close the right eye. Postoperative histopathological examination confirmed the RCCEP diagnosis. Since treatment with camrelizumab and capecitabine was effective, this regimen was continued postoperatively. Although the unresected nodules persisted and new nodules appeared, the patient’s daily life was unaffected. The RCCEP spontaneously resolved after the patient completed 1 year of treatment.

### Potential mechanism of camrelizumab-induced RCCEP and comparison with other capillary hemangiomas

The mechanism by which camrelizumab triggers RCCEP is currently unclear. The predominant theory is that skin capillary endothelial cells exhibit overly active immune responses. RCCEP is histopathologically characterized by enhanced capillary endothelial cell proliferation and numerous mitotic figures. The molecular mechanism of camrelizumab may be that it activates CD4+ T lymphocytes, thereby increasing interleukin-4 levels in T helper 2 cytokines. This subsequently stimulates CD163+ M2 macrophage differentiation and promotes capillary endothelial cell proliferation by releasing vascular endothelial growth factor (VEGF) A (11, 15). Camrelizumab may also induce RCCEP by causing VEGF receptor-2-induced activation of vascular endothelial cell proliferation (16). These proposed mechanisms offer several potential targets for RCCEP prevention.

RCCEP can be classified as a cherry hemangioma (CH), both grossly and histopathologically (17, 18). CHs are the most prevalent form of acquired cutaneous vascular hyperplasia and are more common in older adults; the most frequently affected sites are the trunk and upper extremities (19, 20). CH etiology is attributed to gene mutations, chemical exposure, and viral infection (19). Gene mutation studies have focused on GNAQ, GNA11, and GNA14 (21, 22). Moreover, evidence suggests that VEGFR2 mutations can cause CHs, although the specific mechanism has not been elucidated (23, 24). CH has distinct clinical and histopathological features, although it is not included in the most recent edition of the International Society for the Study of Vascular Anomalies classification of vascular anomalies (25). The early stage of a CH is usually characterized by a flat red spot that gradually enlarges and becomes a red, blue, or purple papule. Histopathological investigations have revealed that CHs consist of lobulated, small-to-mildly dilated, thin-walled vessels with various sized lumens lined with a single layer of endothelial cells (21). These lesions are typically asymptomatic and do not require specific management. Effective treatment methods for CHs can also be used to treat RCCEP.

Infantile hemangioma (IH), also known as infantile capillary hemangioma or strawberry hemangioma, is a benign lesion commonly found on the head, neck, trunk, and extremities. Most of these lesions resolve spontaneously (26). IH growth can be divided into three stages: rapid vascular endothelial cell proliferation, decreased vascular endothelial cell proliferation, and replacement of vascular tissue with fibrofatty tissue (27). Oral propranolol administration, laser therapy, and surgery are the most common clinical treatment options for IH (28). However, the pathogenesis of IH is unclear. The current mainstream view is that pluripotent stem cells respond abnormally to stimuli, such as hypoxia and the renin-angiotensin system (27). As with CH, GNAQ, GNA11, and GNA14 mutations may also cause IH (29–31). Furthermore, gene mutations may interfere with the VEGF A signaling pathway (32, 33). VEGF receptor-2 is the receptor for VEGF A, and some patients with IH have VEGFR2 mutations (34, 35).

Pyogenic granuloma (PG), which is more accurately termed lobular capillary hemangioma, is an acquired benign lesion that occurs in tissues such as the skin and mucous membranes (36, 37). Chronic mild irritation, hormonal imbalances, and drug influences are considered the main PG etiologies (38–40). Cutaneous PG manifests as painless, red, and fleshy nodules that closely resemble RCCEP. Histologically, PG consists of clusters of proliferating capillaries arranged in a lobular structure (41, 42). Current evidence attributes its pathogenesis to effects on the upstream mediator gene, BRAF, on the mitogen-activated protein kinase pathway (43, 44). Although some PGs resolve spontaneously, most require treatment. Treatments include surgical resection, cryotherapy, laser therapy, and imiquimod cream. Among these treatments, surgical resection is the most effective and results in the lowest recurrence rate (37, 45).

### Prevention and treatment of RCCEP caused by camrelizumab

Apatinib has successfully lowered the incidence of RCCEP (46–49). Apatinib is a tyrosine kinase inhibitor that selectively inhibits VEGF receptor-2 (50, 51) and inhibits VEGF-induced endothelial cell migration and proliferation, thereby preventing new blood vessel formation in the tumor tissue. Therefore, the combination of apatinib and camrelizumab may prevent RCCEP development by inhibiting capillary endothelial cell proliferation.

Many studies have shown that patients receiving camrelizumab combined with chemotherapy have better progression-free and overall survival rates than those of patients receiving chemotherapy alone (52–57). Camrelizumab and chemotherapy combined can achieve greater clinical benefits in patients with advanced NPC (58, 59). Camrelizumab combined with chemotherapy can also reduce the risk of RCCEP. Fang et al. reported that camrelizumab administration alone in patients with NPC resulted in a RCCEP incidence of 88% (82/93), compared with only 22% (5/23) when camrelizumab was administered in combination with gemcitabine and cisplatin (58).

By preserving immune activity, anti-programmed cell death protein 1 therapy suppresses tumors over the long term. An overactivated immune system may cause irAEs. Improvements in illness prognosis and the emergence of irAEs represent two sides of the same coin. The clinician is responsible for adjusting the medication regimen according to the clinical situation and intervening if adverse reactions occur. As previously stated, most patients with RCCEP do not require special treatment. RCCEP may gradually resolve if camrelizumab is ineffective and subsequently discontinued. If rupture and bleeding occur, the wound surface should be promptly disinfected, and antibacterial drugs should be administered externally if necessary. Therapeutic measures can be taken when RCCEP adversely affects the patients’ daily life. Traditional treatment methods include cryotherapy, electrosurgery, ligation, and surgical resection (19, 60). With the recent development of light therapy, safer and more effective options have become available for the treatment of capillary hemangiomas; nevertheless, these treatments are expensive (61–63). Several types of lasers can be used to treat capillary hemangiomas, including pulsed dye, alexandrite, neodymium-doped yttrium aluminum garnet, copper bromide, krypton, 532-nm diode, and potassium-titanyl-phosphate lasers (63–67). Intense pulsed light therapy can also be used to treat capillary hemangiomas (68). In cases of grade 3 or higher RCCEP, drug therapy should be immediately discontinued to reduce the mortality risk.

## Conclusion

The development of immunotherapeutic drugs has increased the possibilities for cancer treatment. Meanwhile, cancer diagnosis and treatment require ever-increasing levels of cooperation among multiple disciplines, and increased focus on safe and rational drug administration is required by clinicians. Herein, we described a patient with camrelizumab-induced RCCEP in the right lower eyelid. Although this lesion affected the patient’s ability to close the right eye, their prognosis was good after surgery. This case illustrates the importance of considering the therapeutic efficacy versus the risk to maximize the benefit during cancer treatment. In addition, the data obtained in our clinical practice and provided herein can be used as a reference for improved medication regimen guidance.

## Data availability statement

The original contributions presented in the study are included in the article/supplementary material. Further inquiries can be directed to the corresponding author.

## Ethics statement

The studies involving humans were approved by Ethics Committee of the Second Affiliated Hospital of Shantou University Medical College. The studies were conducted in accordance with the local legislation and institutional requirements. The participants provided their written informed consent to participate in this study. Written informed consent was obtained from the individual(s) for the publication of any potentially identifiable images or data included in this article.

## Author contributions

YL: Conceptualization, Data curation, Formal analysis, Methodology, Writing – original draft. YXL: Data curation, Writing – original draft. XZ: Methodology, Supervision, Writing – review & editing. QC: Supervision, Writing – review & editing. ST: Methodology, Supervision, Writing – review & editing. JC: Data curation, Funding acquisition, Methodology, Project administration, Supervision, Writing – review & editing.
